# Supplementary material for: A highly sensitive strategy for monitoring real-time proliferation of targeted cell types in vivo
Source: Nat Commun. 2023 Jun 14;14:3253. doi: 10.1038/s41467-023-38897-5 (PMC10267192; doi:10.1038/s41467-023-38897-5)
Supplement: Supplementary file 1 — Supplementary Information [file 41467_2023_38897_MOESM1_ESM.pdf]

Sugawara et al. Supplementary Figure 1

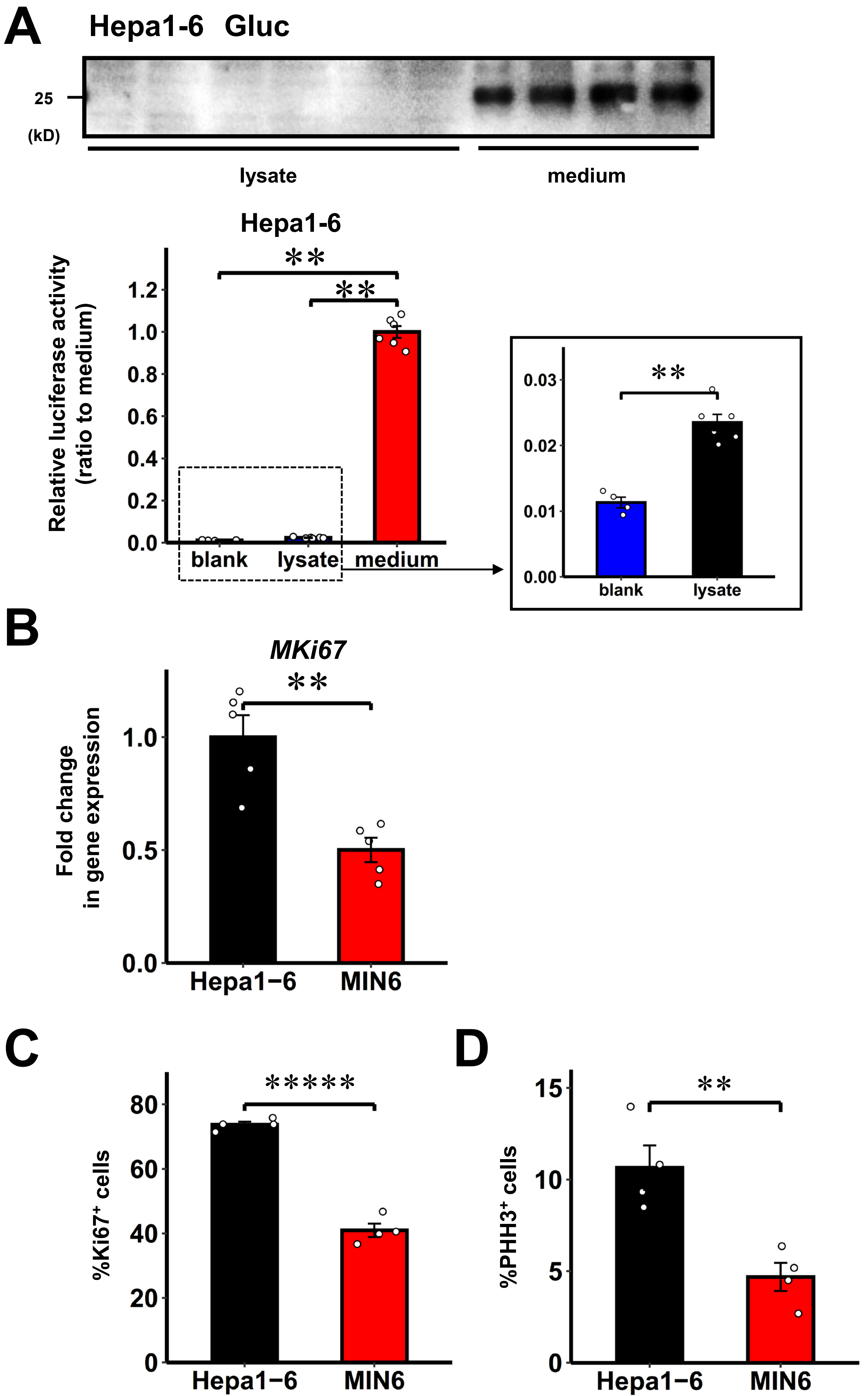

Supplementary Figure 1

Gaussia princeps luciferase are secreted in the culture media and their activities reflect actual cell proliferation status of cultured cell lines.

(A) (Upper panel) Expressions of Gluc in cell lysates or culture media of Cre-adenovirus infected Hepa1-6 cells after 48 h incubation. Culture media were diluted by a factor of 10. (Lower panel) Luciferase activity in cell lysate of Cre-adenovirus infected Hepa1-6 cells relative to that in culture media of these cells. The magnified graph is shown in a framed box with a lowered scale range. (B) Gene expression of mouse Ki67 in Cre-adenovirus infected MIN6 cells. Gene expression in Cre-adenovirus-infected Hepa1-6 cells served as the control. (C) Ki67-positive cell ratios in Cre-adenovirus infected Hepa1-6 and MIN6 cells. (D) PHH3-positive cell ratios in Cre-adenovirus infected Hepa1-6 and MIN6 cells.

Data are presented as means ± SEM. *****$p<0.00005$, **$p<0.01$, assessed by two-sided paired t-test (A), or two-sided unpaired t-test (B-D). (A) n = 6 independent samples for medium, n = 6 independent samples for lysate, n = 4 for blank. Results are representative of two independent experiments. (B) n = 5 independent samples for each group. (C) n = 4 independent samples for each group. (D) n = 4 independent samples for each group. Exact P values are A, P = 6.8E-14 (blank vs. medium), P = 1.9E-14 (lysate vs. medium), P = 8.3E-5 (blank vs. lysate); B, P = 0.0021; C, P = 7.3E-6; D, P = 0.0059. Source data are provided as a Source Data file.

Sugawara et al. Supplementary Figure 2

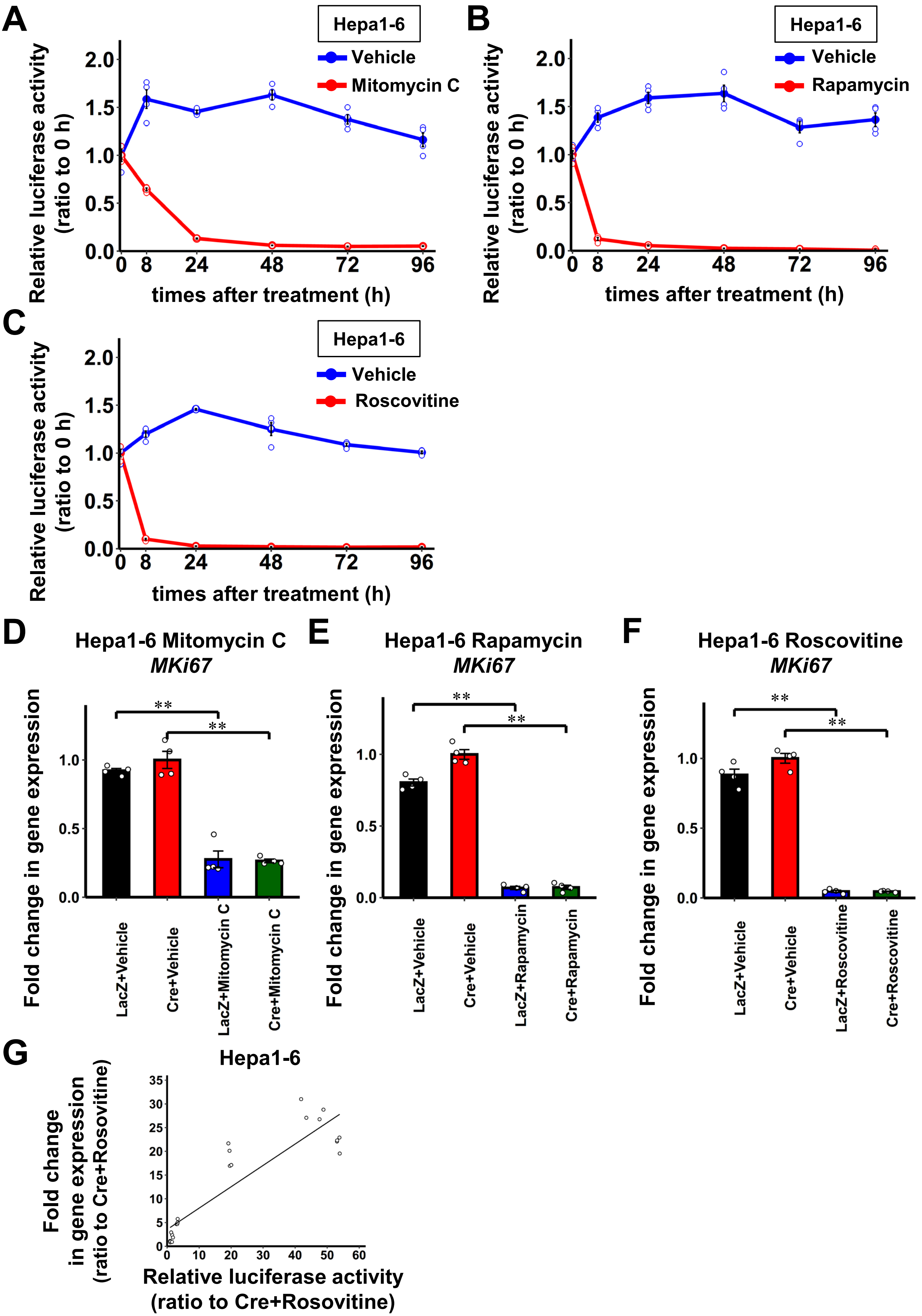

Supplementary Figure 2

Evaluation the cell proliferative status of Cre-adenovirus infected Hepa1-6 cells after treatment with the inhibitors.

(A-C) Time courses of luciferase activity in culture media of Cre-adenovirus infected Hepa1-6 cells after treatment with Mitomycin C (A), Rapamycin (B), or Roscovitine (C) relative to those in culture media before treatment (0 h) (red). Culture media of Cre-adenovirus infected Hepa1-6 cells after treatment with vehicle served as control (blue). (D-F) Gene expression of mouse Ki67 in LacZ-adenovirus infected or Cre-adenovirus infected Hepa1-6 cells after treatment with Mitomycin C (D), Rapamycin (E), or Roscovitine (F) for 24 hours. Gene expression in Cre-adenovirus infected Hepa1-6 cells treated with vehicle served as the control. (G) Linear relationship between Gene expression of mouse Ki67 in Cre-adenovirus infected Hepa1-6 cells after treatment with Mitomycin C, Rapamycin, or Roscovitine and relative luciferase activity in culture media of these cells relative to those in Cre-adenovirus infected Hepa1-6 cells treated with Roscovitine. Open circles indicate Gene expression of mouse Ki67 and relative luciferase activity relative to those in Cre-adenovirus infected Hepa1-6 cells treated with Roscovitine ($r = 0.903$, $P = 1.5E-9$).

Data are presented as means ± SEM. **$p<0.01$, assessed by one-way ANOVA followed by Bonferroni's post hoc test (D-F). Pearson's correlation coefficient (two-sided) was used to determine the correlation (G). n = 4 independent samples for each group. Results are representative of two independent experiments. Exact P values are D, P = 1.8E-6 (LacZ + Vehicle vs. LacZ + Mitomycin C), P = 4.2E-7 (Cre + Vehicle vs. Cre + Mitomycin C); E, P = 1.9E-10 (LacZ + Vehicle vs. LacZ + Rapamycin), P = 1.2E-11 (Cre + Vehicle vs. Cre + Rapamycin); F, P = 3.3E-10 (LacZ + Vehicle vs. LacZ + Roscovitine), P = 6.8E-11 (Cre + Vehicle vs. Cre + Roscovitine). Source data are provided as a Source Data file.

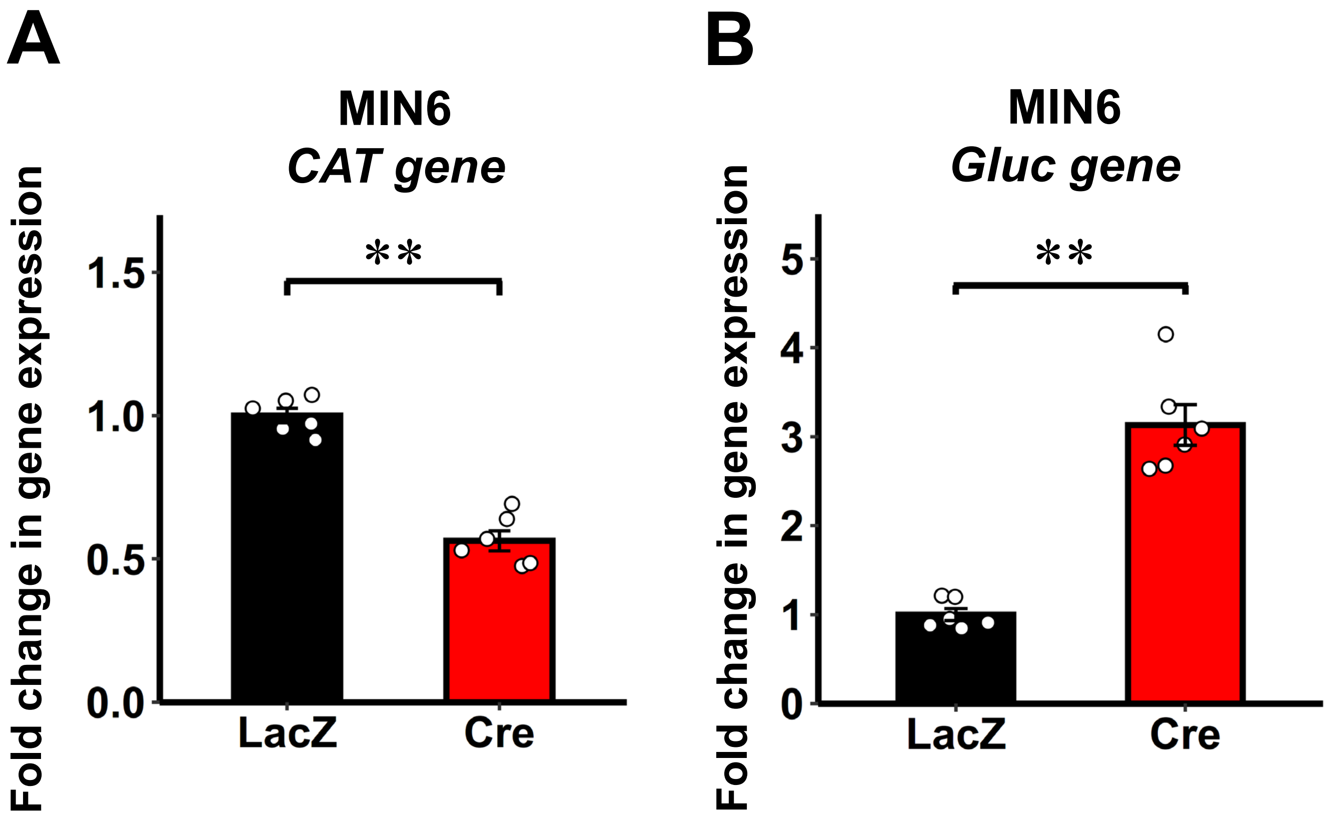
A
MIN6
CAT gene
Fold change in gene expression
1.5
1.0
0.5
0.0
**
LacZ
Cre
B
MIN6
Gluc gene
Fold change in gene expression
5
4
3
2
1
0
**
LacZ
Cre

Supplementary Figure 3

Evaluation of gene expression levels in adenovirus infected MIN6 by quantitative RT-PCR

Expression of the CAT gene (A) or the Gluc gene (B) in Cre-adenovirus infected MIN6 cells. Gene expression in LacZ-adenovirus-infected cells served as the control.

Data are presented as means ± SEM. **$p<0.01$, assessed by two-sided unpaired t-test. n = 6, for each group. Exact P values are A, P = 1.5E-6; B, P = 4.6E-6. Source data are provided as a Source Data file.

A

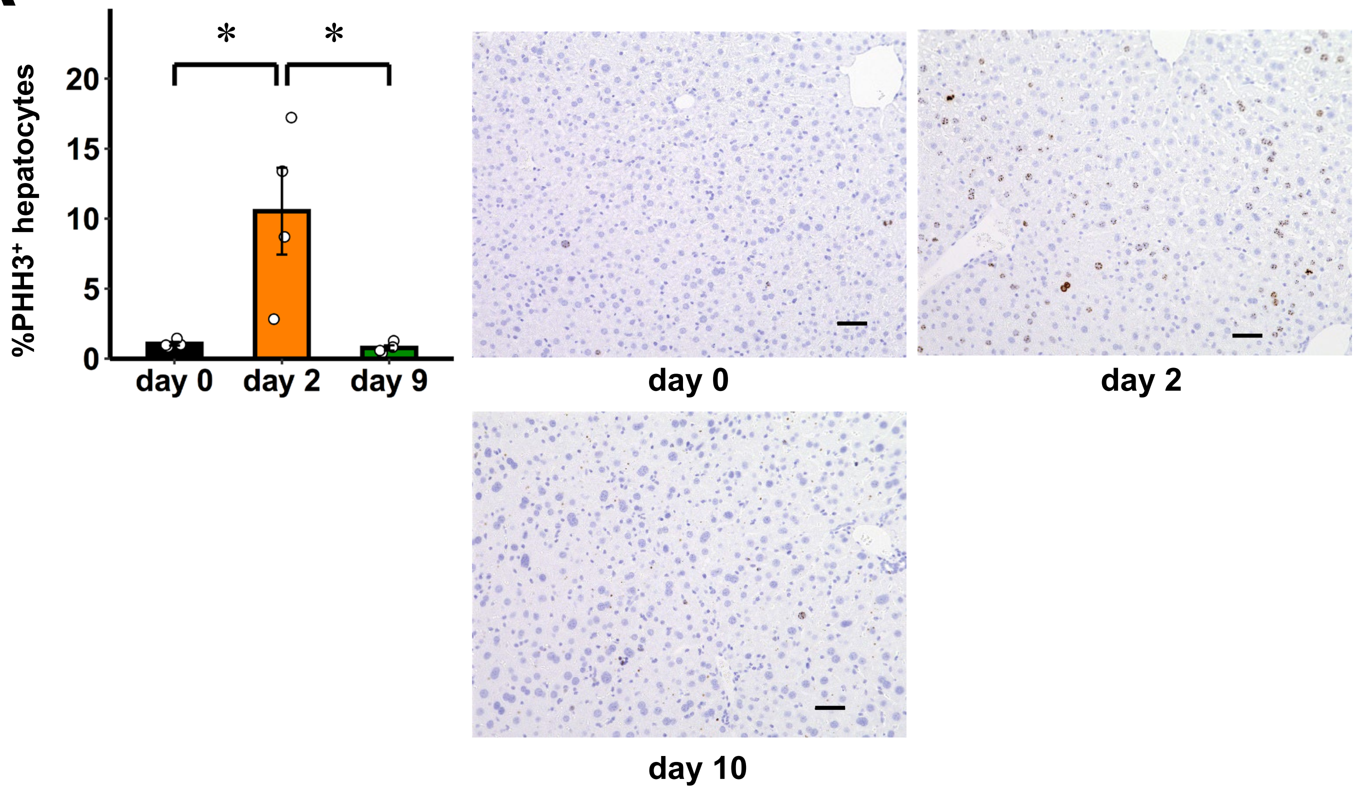
%PHH3⁺ hepatocytes
0
5
10
15
20
*
*
day 0
day 2
day 9
day 0
day 2
day 10

B

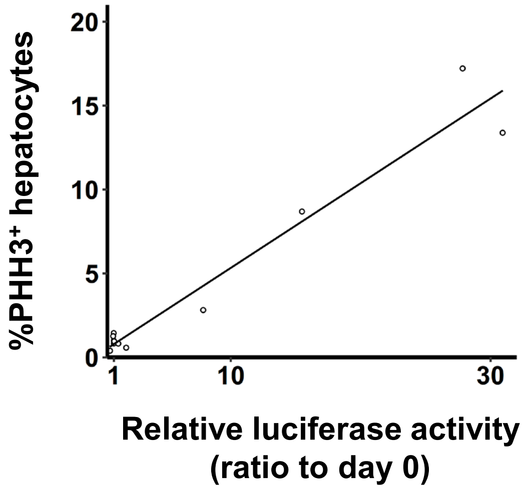
%PHH3⁺ hepatocytes
0
5
10
15
20
1
10
30
Relative luciferase activity
(ratio to day 0)

Supplementary Figure 4

PHH3-positive hepatocyte in situ detection in iLKi67p-Gluc mice after partial hepatectomy

(A) PHH3-positive hepatocyte ratios in 10 weeks old male iLKi67p-Gluc mice on C57BL/6 background on days 0, 2, and 9 after PHx; representative images are shown in the right three panels. Scale bars denote 50 µm. (B) Linear relationship between relative luciferase activity and PHH3-positive hepatocyte ratios. Open circles indicate relative luciferase activity and PHH3-positive hepatocytes in individual 10 weeks old male iLKi67p-Gluc mice after PHx relative to those on day 0 ($r = 0.974$, $P = 8.2E-8$).

Data are presented as means ± SEM. *$p<0.05$, one-way ANOVA followed by Bonferroni's post hoc test (A). Pearson's correlation coefficient (two-sided) was used to determine the correlation (B). n = 4 independent animals for each group, from three independent experiments. Exact P values are A, $P = 0.014$ (day 0 vs. day 2), $P = 0.012$ (day 2 vs. day 9). Source data are provided as a Source Data file.

A

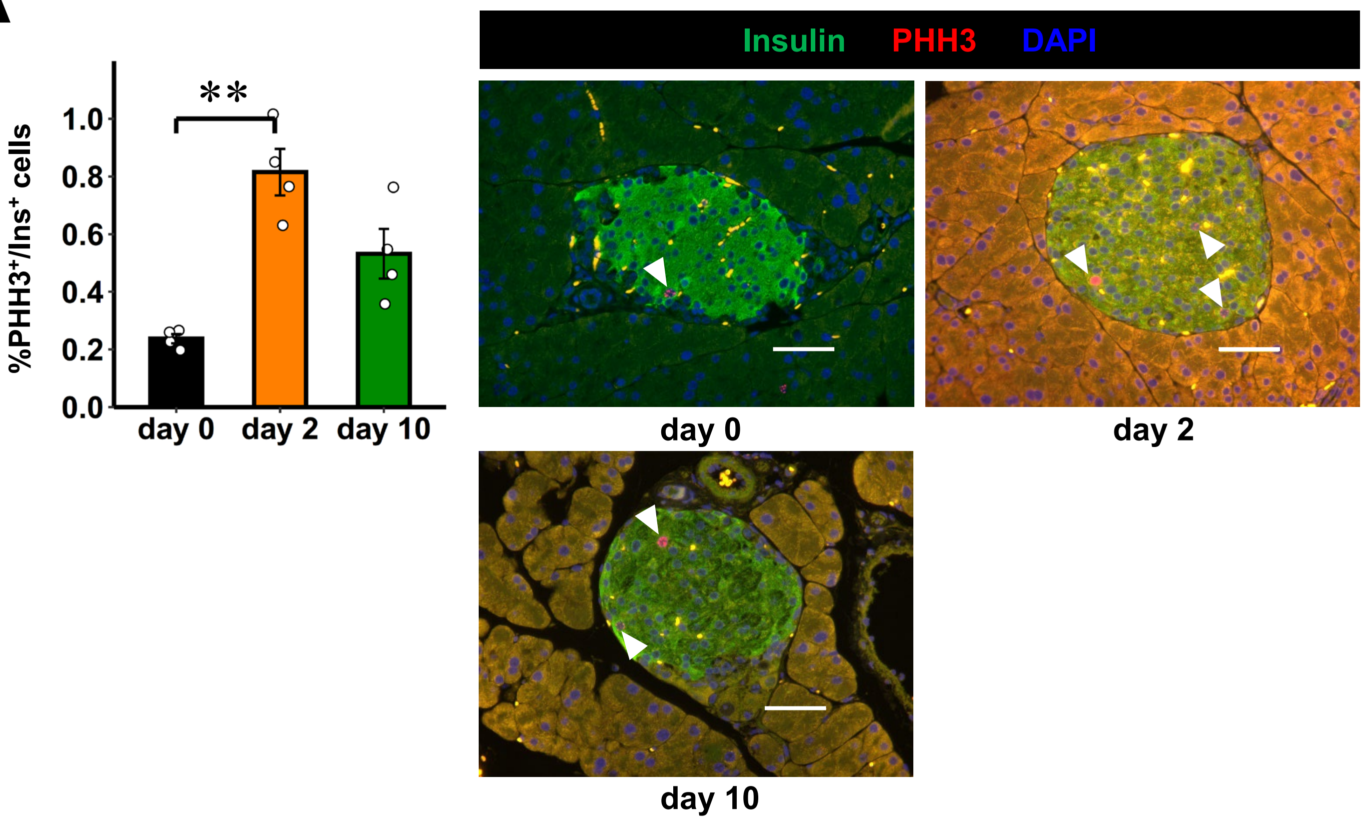

**
1.0
0.8
0.6
0.4
0.2
0.0
%PHH3+/Ins+ cells
day 0
day 2
day 10
Insulin
PHH3
DAPI
day 0
day 2
day 10

B

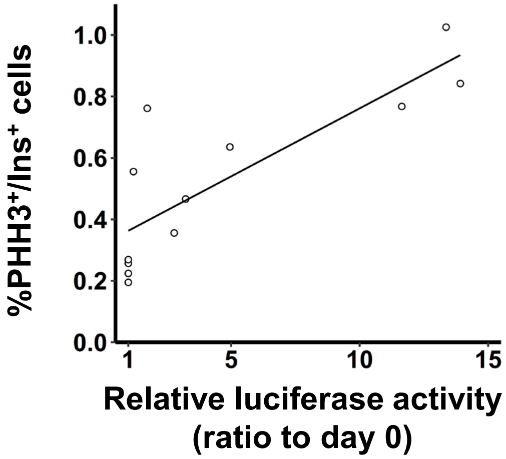

1.0
0.8
0.6
0.4
0.2
0.0
%PHH3+/Ins+ cells
1
5
10
15
Relative luciferase activity
(ratio to day 0)

Supplementary Figure 5

PHH3-positive β-cell in situ detection in iβKi67p-Gluc mice after L-MEK administration

(A) PHH3 and insulin co-positive cell ($PHH3^+/Ins^+$ cells) ratios in insulin-positive cells of 3 months old male iβKi67p-Gluc mice on C57BL/6 background on days 0, 2, and 10 after L-MEK administration relative to those on day 0; representative images are shown in the right three panels. Each arrowhead denotes a $PHH3^+/Ins^+$ cell. Scale bars denote 50 μm. (B) Linear relationship between relative luciferase activity and %$PHH3^+/Ins^+$ cells. Open circles indicate relative luciferase activity and %$PHH3^+/Ins^+$ cells in individual iβKi67p-Gluc mice after L-MEK administration relative to those on day 0 ($r = 0.823$, $P = 0.0010$).

Data are presented as means ± SEM. **$p<0.01$, one-way ANOVA followed by Bonferroni's post hoc test (A). Pearson's correlation coefficient (two-sided) was used to determine the correlation (B). n = 4, for each group. n = 4 independent animals for each group, from four independent experiments. Exact P values are A, P = 6.6E-4 (day 0 vs. day 2). Source data are provided as a Source Data file.

A

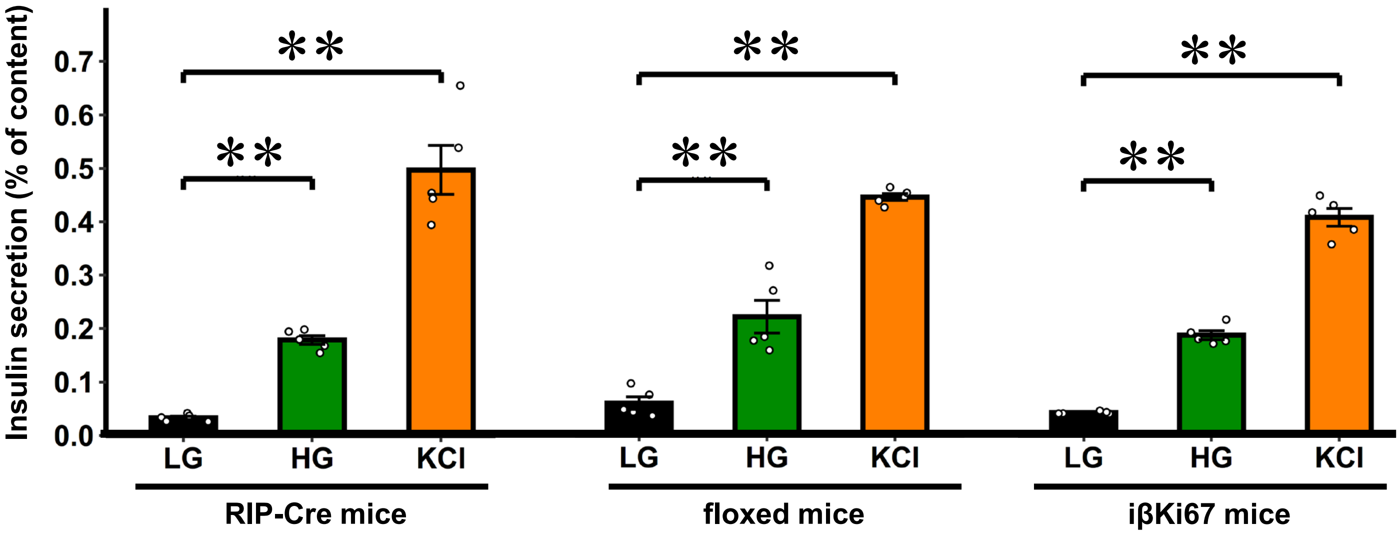
Insulin secretion (% of content)
0.0
0.1
0.2
0.3
0.4
0.5
0.6
0.7
**
LG
HG
KCl
RIP-Cre mice
floxed mice
iβKi67 mice

B

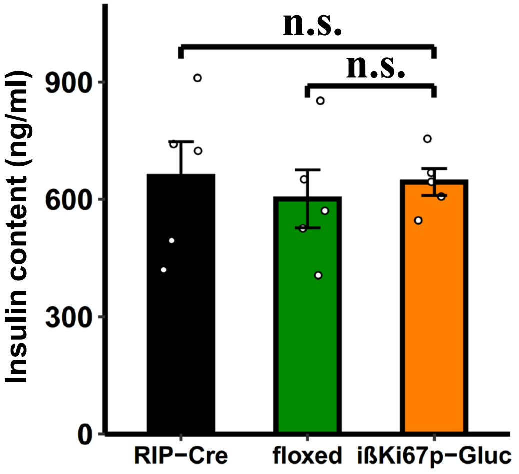
Insulin content (ng/ml)
0
300
600
900
n.s.
RIP−Cre
floxed
ißKi67p−Gluc

Supplementary Figure 6

Islet isolation and insulin secretion study

(A) Insulin secretion, shown as the percentage of insulin content, assessed in 1-hour static incubations in islets isolated from 3 months old male RIP-Cre mice, floxed mice, or iβKi67p-Gluc mice on C57BL/6 background in response to 1.67-, 16.7-mM glucose (LG, HG) or 1.67 mmol/l glucose plus 30 mmol/l KCl (KCl). (B) Total islet insulin content. Data are presented as means ± SEM. ** $p<0.01$, assessed by one-way ANOVA followed by Bonferroni's post hoc test. n.s., not significant. n = 5 independent animals for each group, from three independent experiments. Exact P values are A, P = 0.0072 (RIP-Cre mice; LG vs. HG), P = 1.2E-7 (RIP mice; LG vs. KCl), P = 2.1E-4 (floxed mice; LG vs. HG), P = 2.3E-8 (floxed mice; LG vs. KCl), P = 1.4E-6 (iβKi67p-Gluc mice; LG vs. HG), P = 3.8E-11 (iβKi67p-Gluc mice; LG vs. KCl). Source data are provided as a Source Data file.

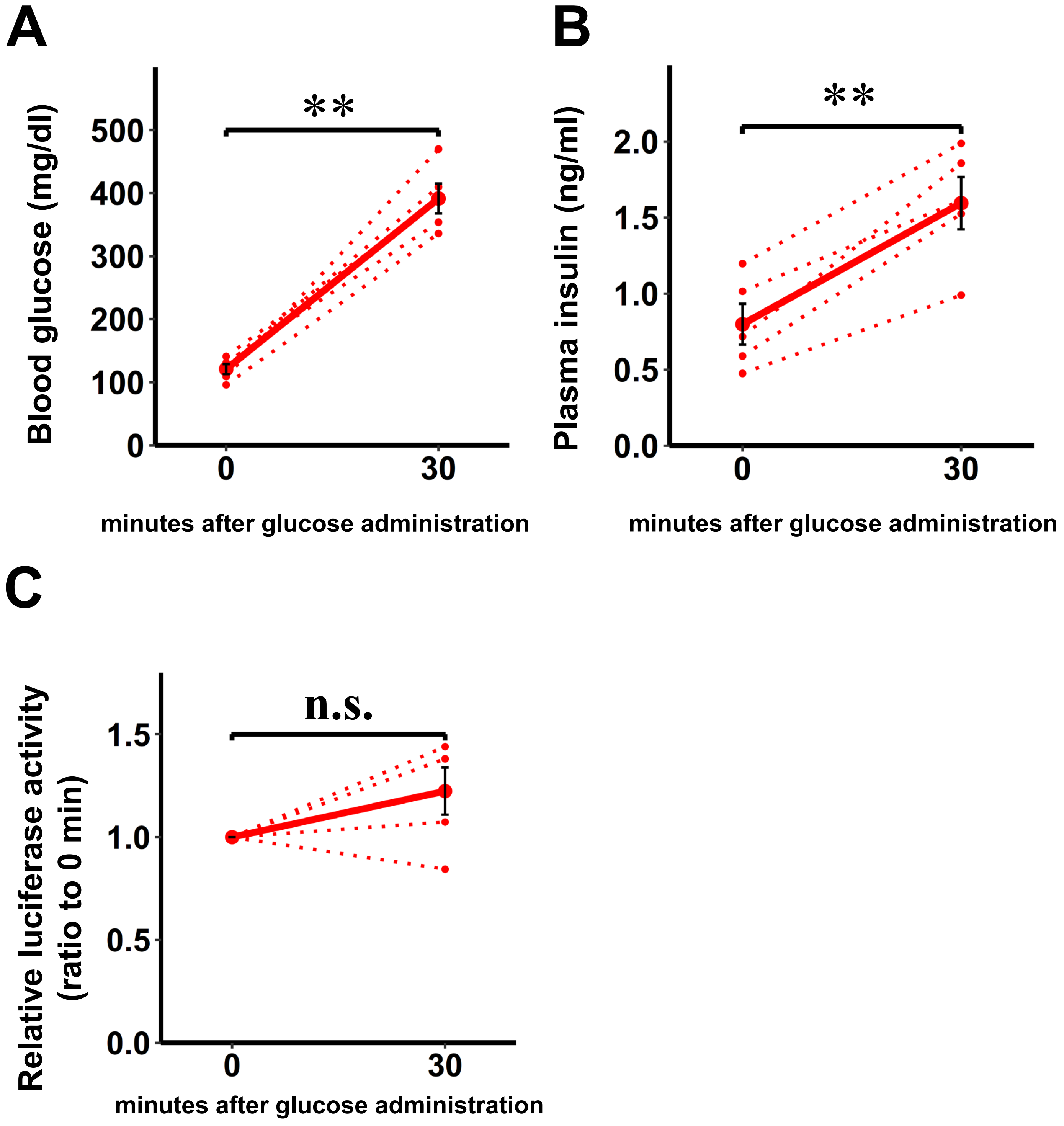
A
Blood glucose (mg/dl)
**
500
400
300
200
100
0
0
30
minutes after glucose administration
B
Plasma insulin (ng/ml)
**
2.0
1.5
1.0
0.5
0.0
0
30
minutes after glucose administration
C
Relative luciferase activity
(ratio to 0 min)
n.s.
1.5
1.0
0.5
0.0
0
30
minutes after glucose administration

Supplementary Figure 7

Glucose tolerance test of iβKi67p-Gluc mice on day 2 after L-MEK administration

Blood glucose level (A), insulin level (B) and luciferase activity (C) in plasma of 3 months old male iβKi67p-Gluc mice on C57BL/6 background 30 minutes after glucose administration. Data are presented as means ± SEM. The values before glucose administration served as controls. Solid lines and dotted lines indicate average and individual values, respectively.

Data are presented as means ± SEM. n.s., not significant, **p<0.01, assessed by two-sided paired t-test. n = 5 independent animals for each group. Exact P values are A, P = 0.00039; B, P= 0.0022. Source data are provided as a Source Data file.

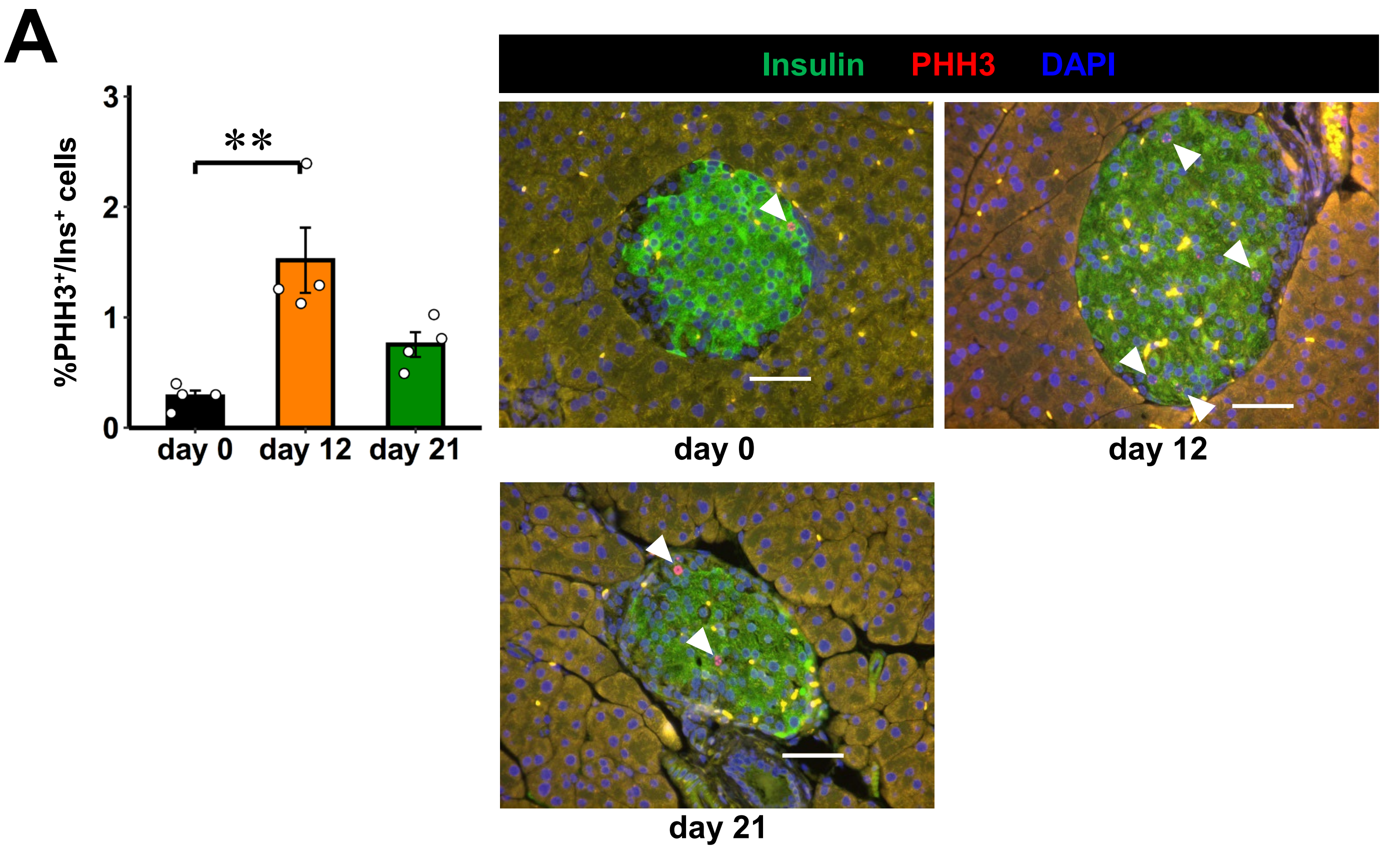
A
%PHH3+/Ins+ cells
3
2
1
0
**
day 0
day 12
day 21
Insulin
PHH3
DAPI
day 0
day 12
day 21

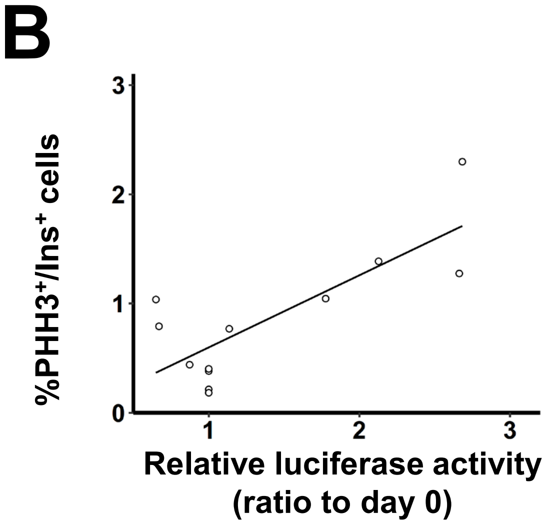
B
%PHH3+/Ins+ cells
3
2
1
0
1
2
3
Relative luciferase activity
(ratio to day 0)

Supplementary Figure 8

PHH3-positive β-cell in situ detection in iβKi67p-Gluc mice during pregnancy

(A) $PHH3^+/Ins^+$ cell ratios in insulin-positive cells of 3 months old female iβKi67p-Gluc mice on C57BL/6 background on days 0, 12, and 21 after mating relative to those on day 0; representative images are shown in the right three panels. Each arrowhead denotes a $PHH3^+/Ins^+$ cell. Scale bars denote 50 μm. (B) Linear relationship between relative luciferase activity and % $PHH3^+/Ins^+$ cells. Open circles indicate relative luciferase activity and % $PHH3^+/Ins^+$ cells in individual iβKi67p-Gluc mice after mating relative to those on day 0 (r = 0.777, P = 0.0030).

Data are presented as means ± SEM. **p<0.01, one-way ANOVA followed by Bonferroni's post hoc test (A). Pearson's correlation coefficient (two-sided) was used to determine the correlation (B). n = 4, for each group. (C, D) n = 4 independent animals for each group, from four independent experiments. Exact P values are A, P = 0.0032 (day 0 vs. day 12). Source data are provided as a Source Data file.

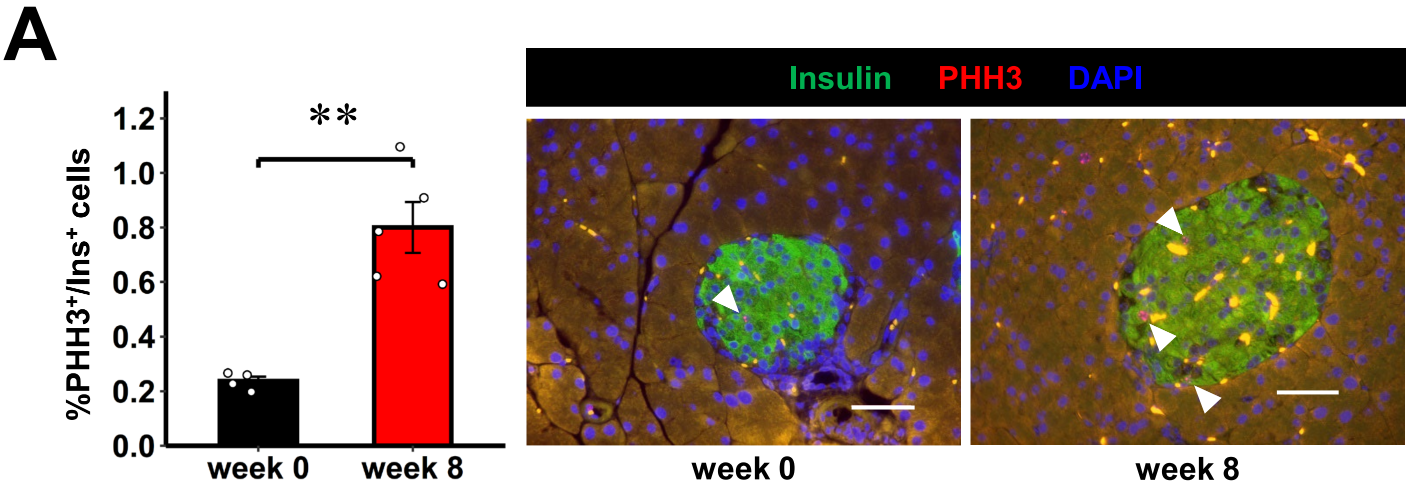
A
Insulin
PHH3
DAPI
**
%PHH3+/Ins+ cells
1.2
1.0
0.8
0.6
0.4
0.2
0.0
week 0
week 8
week 0
week 8

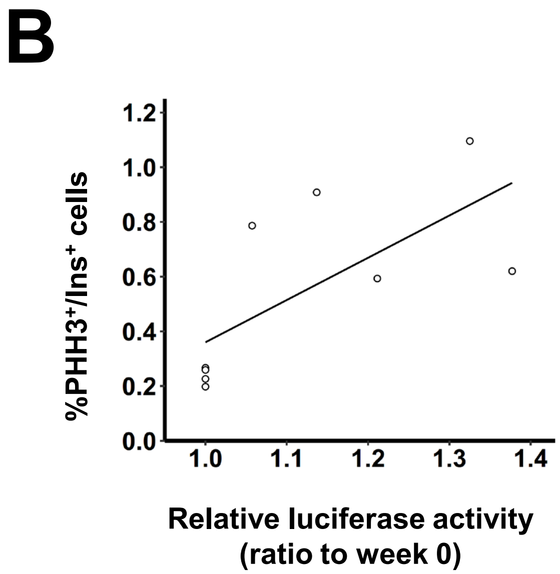
B
%PHH3+/Ins+ cells
1.2
1.0
0.8
0.6
0.4
0.2
0.0
1.0
1.1
1.2
1.3
1.4
Relative luciferase activity
(ratio to week 0)

Supplementary Figure 9

PHH3 positive β-cell in situ detection in iβKi67p-Gluc mice after high fat loading

(A) PHH3 and insulin co-positive cell ($PHH3^+/Ins^+$ cells) ratios in insulin-positive cells of 3 months old male iβKi67p-Gluc mice on C57BL/6 background at weeks 0 and 8 after high-fat loading relative to those on week 0; representative images are shown in the right two panels. Each arrowhead denotes a $PHH3^+/Ins^+$ cell. Scale bars denote 50 μm. (B) Linear relationship between relative luciferase activity and % $PHH3^+/Ins^+$ cells. Open circles indicate relative luciferase activity and % $PHH3^+/Ins^+$ cells in individual iβKi67p-Gluc mice after high fat loading relative to those at week 0 (r = 0.694, P = 0.038)

Data are presented as means ± SEM. **p<0.01, one-way ANOVA followed by Bonferroni's post hoc test (A). Pearson's correlation coefficient (two-sided) was used to determine the correlation (B). n = 4, for each group. (A) n = 5 independent animals for HFD, n = 4 independent animals for NC, from three independent experiments. Exact P values are A, P = 0.0012. Source data are provided as a Source Data file.

A

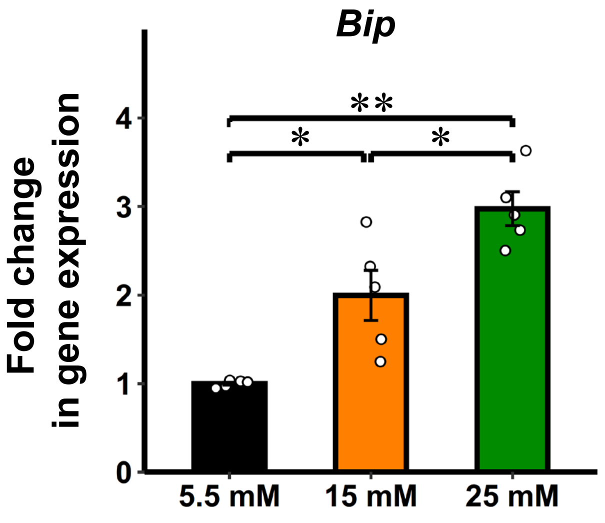
Bip
Fold change
in gene expression
**
*
*
0
1
2
3
4
5.5 mM
15 mM
25 mM

B

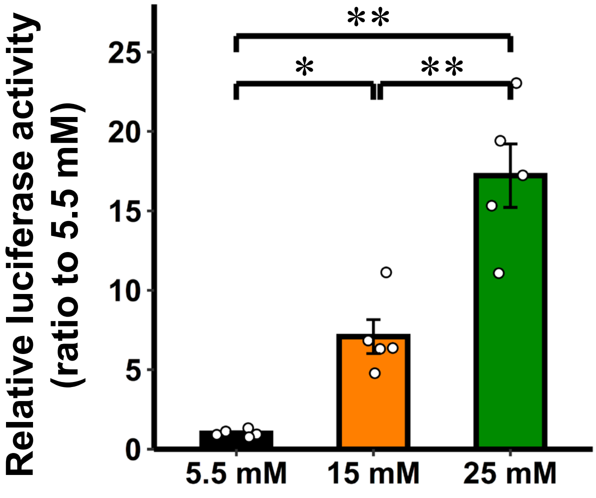
Relative luciferase activity
(ratio to 5.5 mM)
**
*
**
0
5
10
15
20
25
5.5 mM
15 mM
25 mM

C

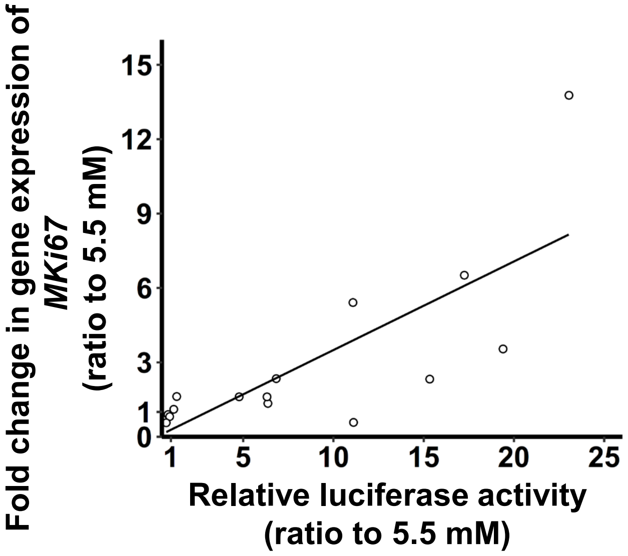
Fold change in gene expression of
MKi67
(ratio to 5.5 mM)
0
1
3
6
9
12
15
1
5
10
15
20
25
Relative luciferase activity
(ratio to 5.5 mM)

Supplementary Figure 10

Ex vivo monitoring of β-cell proliferation using isolated islets from iβKi67p-Gluc mice

(A) Gene expression of Bip in isolated islets from 3 months old male iβKi67p-Gluc mice on C57BL/6 background after stimulation with 15-, or 25-mM glucose. Gene expression in isolated islets cultured with a 5.5 mM glucose concentration served as control. (B) Luciferase activity in the culture media of these islets. Luciferase activity in culture media of isolated islets cultured with a 5.5 mM glucose concentration served as control. (C) Linear relationship between relative luciferase activity and gene expression of mouse Ki67 in these islets. Open circles indicate relative luciferase activity and gene expression of mouse Ki67 in these islets ($r = 0.766$, $P = 0.00087$).

Data are presented as means ± SEM. n.s., not significant, $^{**}p<0.01$, $^{*}p<0.05$, assessed by one-way ANOVA followed by Bonferroni's post hoc test. (A, B). Pearson's correlation coefficient (two-sided) was used to determine the correlation (C). (A) n = 5 independent samples from two independent experiments. Exact P values are A, P = 0.012 (5.5 mM vs. 15 mM), P=3.9E-5 (5.5 mM vs. 25 mM), P = 0.013 (15 mM vs. 25 mM); B, P = 0.020 (5.5 mM vs. 15 mM), P = 4.4E-6 (5.5 mM vs. 25 mM), P = 4.3E-4 (15 mM vs. 25 mM). Source data are provided as a Source Data file.

A

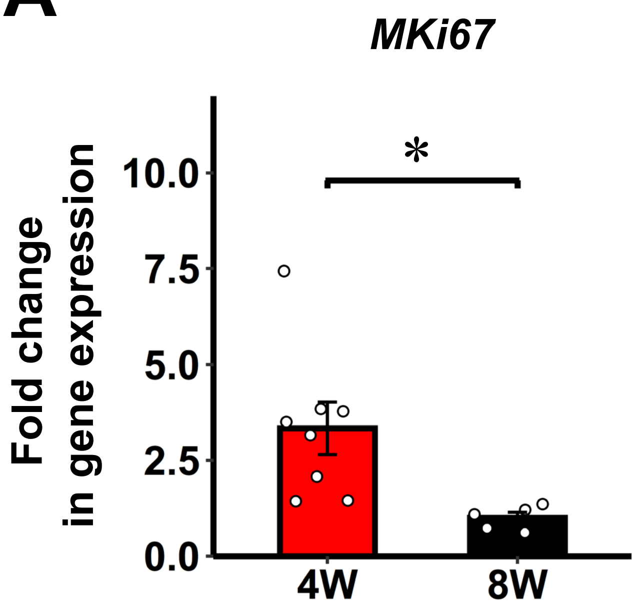
MKi67
Fold change in gene expression
10.0
7.5
5.0
2.5
0.0
*
4W
8W

B

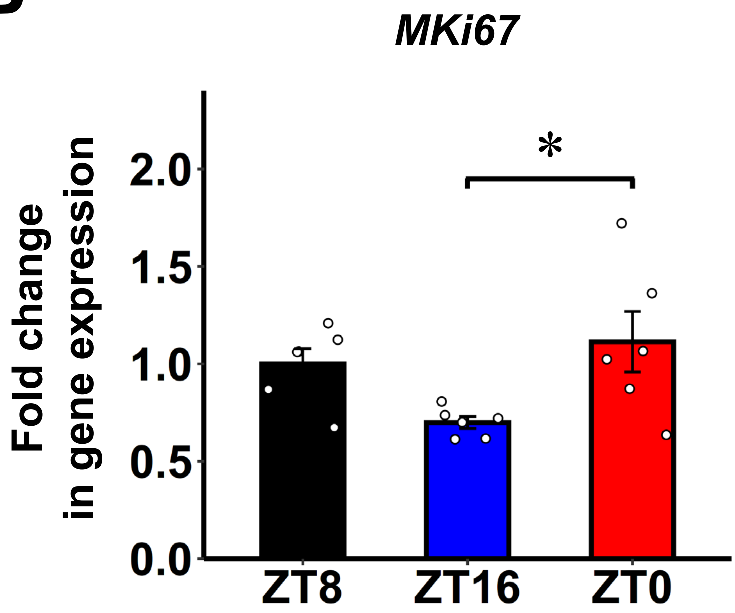
MKi67
Fold change in gene expression
2.0
1.5
1.0
0.5
0.0
*
ZT8
ZT16
ZT0

Supplementary Figure 11

Evaluation of gene expression levels in isolated islets from C57BL/6N mice

(A) Gene expression of mouse Ki67 in isolated islets from 4-week-old male C57BL/6N mice on C57BL/6 background. Gene expression in isolated islets from 8-week-old male C57BL/6N mice served as controls. (B) Gene expression of mouse Ki67 in the islets isolated at ZT 16 or 0 from 4-week-old male C57BL/6N mice. Gene expression in the islets isolated at ZT 8 served as controls.

Data are presented as means ± SEM. *p<0.05, assessed by two-sided unpaired t-test (A), or one-way ANOVA followed by Bonferroni's post hoc test (B). (A) n = 8 independent samples for 4W, n = 5 independent samples for 8W, from two independent experiments. (B) n = 6 independent samples for each group from three independent experiments. Exact P values are A, P = 0.023; B, P = 0.036. Source data are provided as a Source Data file.

A

Supplementary Figure 1A

Hepa1-6 Gluc

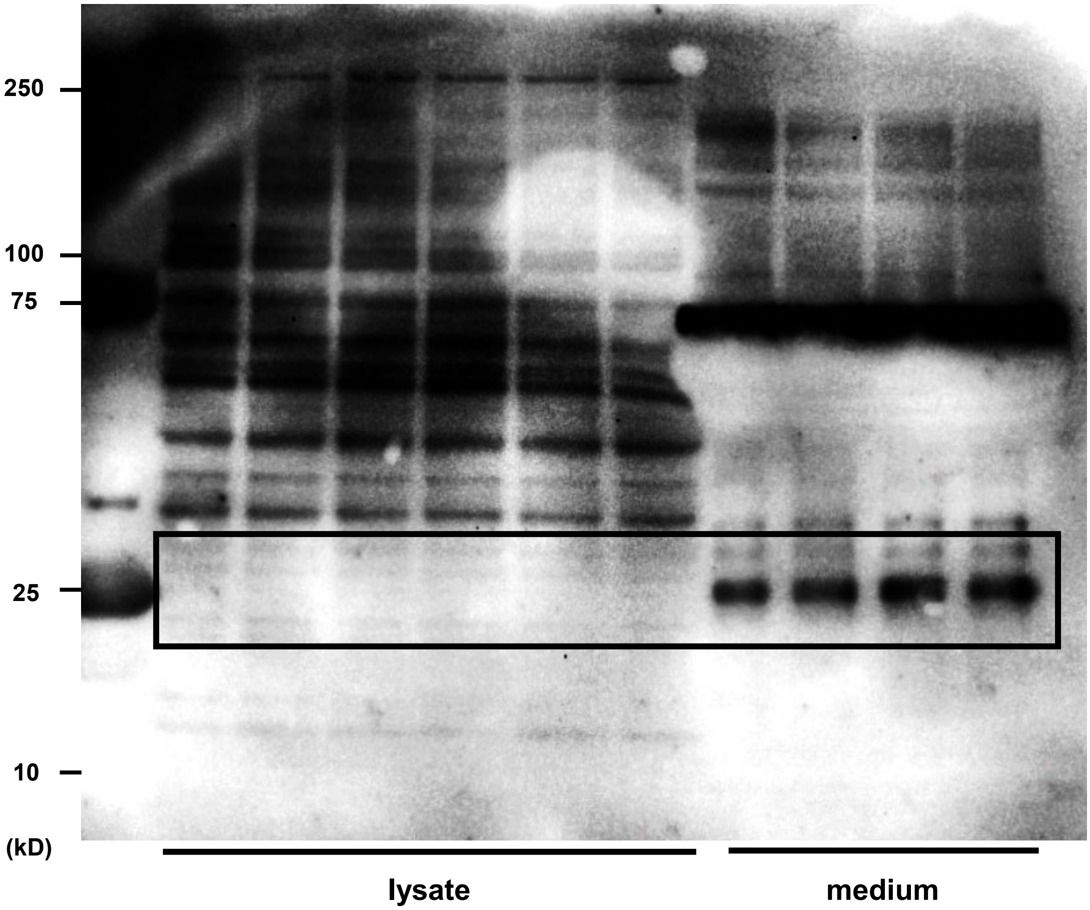

Supplementary Figure 12

Uncropped scans of the immunoblots for Supplementary Figures 1A

	forward (5' to 3')	reverse (5' to 3')
Ki67p	CCCTCGGTTCGAGGTTCCCAACGA	ATCCGGCCCGCAAGGCCACTTGT
Mouse ki67	AGTCTCTGGAGAGTCTGATGTTA	ACTTCTTGGTGCATACAATGTC
Mouse Actb	GATGCCCTGAGGCTCTT	TGTGTTGGCATAGAGGTCTTTAC
Mouse CAT	CAACCTATGGAACTGATGAATGG	CTCATCATCACTAGATGGCATTTC
Mouse gaussia luciferase	ATCGTCGACATTCCTGAGATTC	GGTCAGAACACTGCACGTTG
Mouse Bip	GACATTTGCCCCAGAAGAAA	CTCATGACATTCAGTCCAGCA

Supplementary Table 1

Sequences of the quantitative RT-PCR primers used in this study.
