# Supplementary material for: A highly sensitive strategy for monitoring real-time proliferation of targeted cell types in vivo
Source: Nat Commun. 2023 Jun 14;14:3253. doi: 10.1038/s41467-023-38897-5 (PMC10267192; doi:10.1038/s41467-023-38897-5)
Supplement: Supplementary file 3 — Reporting Summary [file 41467_2023_38897_MOESM3_ESM.pdf]

nature portfolio

Corresponding author(s): Junta Imai
Last updated by author(s): May 16, 2023

Reporting Summary

Nature Portfolio wishes to improve the reproducibility of the work that we publish. This form provides structure for consistency and transparency in reporting. For further information on Nature Portfolio policies, see our Editorial Policies and the Editorial Policy Checklist.

Statistics

For all statistical analyses, confirm that the following items are present in the figure legend, table legend, main text, or Methods section.

n/a	Confirmed	
☐	☒	The exact sample size (*n*) for each experimental group/condition, given as a discrete number and unit of measurement
☐	☒	A statement on whether measurements were taken from distinct samples or whether the same sample was measured repeatedly
☐	☒	The statistical test(s) used AND whether they are one- or two-sided<br>*Only common tests should be described solely by name; describe more complex techniques in the Methods section.*
☐	☒	A description of all covariates tested
☐	☒	A description of any assumptions or corrections, such as tests of normality and adjustment for multiple comparisons
☐	☒	A full description of the statistical parameters including central tendency (e.g. means) or other basic estimates (e.g. regression coefficient) AND variation (e.g. standard deviation) or associated estimates of uncertainty (e.g. confidence intervals)
☐	☒	For null hypothesis testing, the test statistic (e.g. *F*, *t*, *r*) with confidence intervals, effect sizes, degrees of freedom and *P* value noted<br>*Give P values as exact values whenever suitable.*
☐	☒	For Bayesian analysis, information on the choice of priors and Markov chain Monte Carlo settings
☐	☒	For hierarchical and complex designs, identification of the appropriate level for tests and full reporting of outcomes
☐	☒	Estimates of effect sizes (e.g. Cohen's *d*, Pearson's *r*), indicating how they were calculated

Our web collection on statistics for biologists contains articles on many of the points above.

Software and code

Policy information about availability of computer code

Data collection	Gluc activity was measured using a plate luminometer (Fluoroscan Ascent® FL, Thermo Fisher Scientific) . cDNA synthesized from 100 ng of total RNA with a QuantiTect Reverse Transcription Kit (QIAGEN) was evaluated with a real-time PCR quantitative system (Light Cycler software; Roche Diagnostics, Mannheim, Germany). Gluc imaging was performed using a cooled CCD camera (IVIS SPECTRUM, PerkinElmer, Waltham, MA, USA)
Data analysis	Microsoft Excel was used for statistical analyses.

For manuscripts utilizing custom algorithms or software that are central to the research but not yet described in published literature, software must be made available to editors and reviewers. We strongly encourage code deposition in a community repository (e.g. GitHub). See the Nature Portfolio guidelines for submitting code & software for further information.

Data

Policy information about availability of data

All manuscripts must include a data availability statement. This statement should provide the following information, where applicable:

- Accession codes, unique identifiers, or web links for publicly available datasets
- A description of any restrictions on data availability
- For clinical datasets or third party data, please ensure that the statement adheres to our policy

Source data are provided with this paper.

Research involving human participants, their data, or biological material

Policy information about studies with human participants or human data. See also policy information about sex, gender (identity/presentation), and sexual orientation and race, ethnicity and racism.

Reporting on sex and gender	Not applicable
Reporting on race, ethnicity, or other socially relevant groupings	Not applicable
Population characteristics	Not applicable
Recruitment	Not applicable
Ethics oversight	Not applicable

Note that full information on the approval of the study protocol must also be provided in the manuscript.

Field-specific reporting

Please select the one below that is the best fit for your research. If you are not sure, read the appropriate sections before making your selection.

[x] Life sciences [ ] Behavioural & social sciences [ ] Ecological, evolutionary & environmental sciences

For a reference copy of the document with all sections, see nature.com/documents/nr-reporting-summary-flat.pdf

Life sciences study design

All studies must disclose on these points even when the disclosure is negative.

Sample size	Sample size was approximately based on previous studies (PMID: 19023081, 29208957, 30546054) in the field, pilot experiments, and reviewer's suggestion. A reasonable sample size was estimated to perform valid statistical analysis and to ensure the reproducibility of the results.
Data exclusions	No data were excluded from analyses.
Replication	More than two independent experiments were performed independently in every experiments to ensure that the experimental results were reliable. Detailed information on the matter can be found in figure legends.
Randomization	Statistical analyses were performed assuming a normal distribution in all experiments.
Blinding	Blinding was not required as the same analysis was adopted for all experimental conditions during data acquisition and analysis.

Reporting for specific materials, systems and methods

We require information from authors about some types of materials, experimental systems and methods used in many studies. Here, indicate whether each material, system or method listed is relevant to your study. If you are not sure if a list item applies to your research, read the appropriate section before selecting a response.

Materials & experimental systems

n/a	Involved in the study	
[ ]	[x]	Antibodies
[ ]	[x]	Eukaryotic cell lines
[x]	[ ]	Palaeontology and archaeology
[ ]	[x]	Animals and other organisms
[x]	[ ]	Clinical data
[x]	[ ]	Dual use research of concern
[x]	[ ]	Plants

Methods

n/a	Involved in the study	
[x]	[ ]	ChIP-seq
[x]	[ ]	Flow cytometry
[x]	[ ]	MRI-based neuroimaging

Antibodies

Antibodies used	1) anti-Gaussia luciferase, clone 401P, NanoLight Technologies, dilution rate 1:1000;<br>2) horseradish peroxidase-conjugated antibody, clone NA9340, GE Healthcare, dilution rate 1:1000;<br>3) anti-Ki67, ab15580, Abcam, dilution rate 1:1000;

	4) anti-PHH3, clone 9701, Cell Signaling Technology, dilution rate 1:200;<br>5) Alexa Fluor 594 conjugated-secondary antibody, clone 111-585-144, Jackson ImmunoResearch Laboratories, diution rate 1:500;<br>6) Alexa Fluor 594 conjugate-secondary antibody, clone 8889, Cell Signaling Technology, dilution rate 1:500;<br>7)anti-Ki67, clone 12202, lot 6, Cell Signaling Technology, dilution rate 1:400;<br>8) HRP-labeled antibody, clone 8114, Cell Signaling Technology, dilution rate 1:500;<br>9)anti-insulin, clone IR002, Agilent Technologies, without dilution;<br>10) Alexa Fluor 488 conjugated antibody, clone ab150117, Abcam, dilution rate 1:200;
Validation	Validation statements of all antibodies are on the manufacture's websites.

Eukaryotic cell lines

Policy information about cell lines and Sex and Gender in Research

Cell line source(s)	Hepa 1-6 cells were purchased from ATCC. MIN6-cells were provided from Prof. Junichi Miyazaki (Osaka University).
Authentication	Hepa 1-6 cell ell lines were authenticated by PCR assays with species-specific primers by vendors. No further authentication was performed for Hepa1-6 cells. MIN-6 cells were authenticated by the provider and were further authenticated by morphology and by functional analyses of insulin secretion by the authors.
Mycoplasma contamination	Hepa 1-6 cells and MIN6 cells were not tested for mycoplasma contamination.
Commonly misidentified lines (See ICLAC register)	No commonly misidentified cell lines were used in this study.

Animals and other research organisms

Policy information about studies involving animals; ARRIVE guidelines recommended for reporting animal research, and Sex and Gender in Research

Laboratory animals	alubumin-Cre-ER;Ki67p-LSL-Gluc mice: male, 8 weeks of age. RIP-CreER; KI67p-LSL-Gluc mice: male and female, 8 weeks of age. RIP-Cre; Ki67p-LSL-Gluc mice: male, 8weeks of age. C57BL/6N mice: male, 8 weeks of age.
Wild animals	No wild animals were used in this study.
Reporting on sex	Both male and female mice were used in this study.
Field-collected samples	No field collected samples were used in this study.
Ethics oversight	Animal studies were conducted in accordance with the Tohoku University institutional guidelines. Ethics approval was obtained from the Institutional Animal Care and Use Committee of the Tohoku University Environmental & Safety Committee.

Note that full information on the approval of the study protocol must also be provided in the manuscript.
